# Molecular Cytogenetic Characterization of Novel Wheat-rye T1RS.1BL Translocation Lines with High Resistance to Diseases and Great Agronomic Traits

**DOI:** 10.3389/fpls.2017.00799

**Published:** 2017-05-15

**Authors:** Tianheng Ren, Zongxiang Tang, Shulan Fu, Benju Yan, Feiquan Tan, Zhenglong Ren, Zhi Li

**Affiliations:** ^1^Agronomy College, Sichuan Agricultural UniversitySichuan, China; ^2^College of Life Science, Sichuan Agricultural UniversitySichuan, China

**Keywords:** wheat, rye, translocation, disease resistance, agronomic traits, chromosome mutation

## Abstract

Rye has been used worldwide as a source for the genetic improvement of wheat. In this study, two stable wheat-rye primary T1RS.1BL translocation lines were selected from the progeny of the crossing of the wheat cultivar Mianyang11-1 and a Chinese local rye variety, Weining. These two novel translocation lines were identified by molecular cytogenetic analysis. PCR results, multi-color fluorescence *in situ* hybridization (MC-FISH), and acid polyacrylamide gel electrophoresis (A-PAGE) indicated that both new translocation lines harbor a pair of T1RS.1BL translocation chromosomes, and have been named RT828-10 and RT828-11, respectively. The cytogenetic results also indicated that the pSc119.2 signals of 5AL were absent in both lines along with the pSc119.2 signals of 4AL of RT828-11. When inoculated with different stripe rust and powdery mildew isolates, both lines expressed high resistance to *Puccinia striiformis* f. sp. *tritici* and *Blumeria graminis* f. sp. *tritici* pathotypes, which are prevalent in China and are virulent on *Yr9 and Pm8*. The line RT828-11 also exhibited excellent agronomic traits in the field. The present study indicates that this rye variety may carry untapped variations that could potentially be used for wheat improvement.

## Introduction

Common wheat (*Triticum aestivum* L.) is 1 of the most important crops in the world. For higher yield and better resistance to diseases, many useful genes or factors have been introduced into wheat through chromosome translocation or substitution from many different relative genera (Molnár-Láng et al., 2014; Feldman and Levy, 2015) such as *Pm53, Yr40*, and *Yr42* from *Aegilops* spp. (Marais et al., 2009; Kuraparthy et al., 2009; Petersen et al., 2015), *Pm40* and *Yr50* from *Thinopyrum* spp. (Luo et al., 2009; Liu et al., 2013), *Pm21* from *Haynaldia villosa* (Chen et al., 1995), *YrSn0096* from *Leymus mollis* (Bao et al., 2012), and *Pm2b* from *Agropyron* (Ma et al., 2015). However, rye (*Secale cereal* L.) is the most important and valuable related species for the improvement of wheat genetics (Schlegel and Korzun, 1997; Rabinovich, 1998; Lelley et al., 2004; Ren et al., 2012). Since the 1950s, the 1RS chromosome arm was introduced into common wheat from the German rye variety ‘Petkus’ through a rye-wheat T1RS.1BL translocation line (Mettin et al., 1973; Schlegel and Korzun, 1997). Many resistant genes of rye were transferred into wheat, such as *Yr9, Pm8, Lr26, and Sr31* (Mago et al., 2005; Ren et al., 2009). Moreover, the 1RS chromosome arm also harbors several genes could enhance the yield potential and wide range of environmental adaptability of wheat (Kumlay et al., 2003; Ren et al., 2012; Howell et al., 2014) Therefore, the rye-wheat 1RS.1BL translocation was used worldwide in wheat breeding programs (Rabinovich, 1998). However, the significant weakness of T1RS.1BL lines is its narrow genetic base, which is due to its single origin from Petkus rye (Baum and Appels, 1991; Schlegel and Korzun, 1997; Lelley et al., 2004; Ren et al., 2012).

Stripe rust and powdery mildew, which are caused by *Puccinia striiformis* f. sp. *tritici* (*Pst*) and *Blumeria graminis* f. sp. *tritici* (*Bgt*), respectively, are usually considered to be devastating diseases of wheat in cooler areas (Ren et al., 2009). Since the 1990s, due to its single origin, the 1RS chromosome arm derived from Petkus rye has not provided protection against the prevalence of virulent pathogens (Shi et al., 2001; Ren et al., 2009). So far, only a few other sources of 1RS were transferred into wheat (Ko et al., 2002; Mater et al., 2004; Ren et al., 2009, 2012; Molnár-Láng et al., 2010; Yang et al., 2014; Li et al., 2016a,b; Qi et al., 2016), and not every new translocation line could be used in wheat breeding programs (Ren et al., 2012). For more efficient use of the T1RS.1BL translocation in wheat breeding, Ren et al. (2012) suggested introduce a large amount of new genetic variation from many different rye sources into wheat.

In previous studies, polyploidization or chromosome translocation were considered as factors to induce the genome mutation or evolution (Ozkan et al., 2001; Tang et al., 2009; Li et al., 2016a). In the present study, we reported 2 new primary T1RS.1BL translocation lines which were developed from the crossbreeding of the wheat cultivar Mianyang11-1 (MY11-1) and the local Chinese rye variety Weining. We named these 2 lines RT828-10 and 828-11. Both lines were identified by FISH and molecular analysis, and showed high resistance to stripe rust and powdery mildew. Furthermore, these 2 lines could be excellent materials for use in wheat breeding programs.

## Materials and Methods

### Plant Materials

Weining rye is a Chinese local rye variety collected from southwestern China. The common wheat cultivar Mianyang11 (MY11) was released in 1981, and is widely grown in southwestern China. MY11 contains the *kr1* gene, and therefore could be easily crossed with rye. A selfing line of MY11, which was named as MY11-1, was used in this study. Seeds of MY11-1 used for the crossing were produced by a single spike descent across several generations to create pure genetic stocks. The F1 seedlings of MY11-1 x Weining were soaked in 0.05% colchicine + 3% dimethyl sulfoxide for 8 h to produce the amphidiploid (C1). Then, the C1 plants were backcrossed to MY11-1 to produce monosomic wheat-rye addition lines. A 1R monosomic addition line 98-828 (2n = 43 = 42W+1’1R) was selected and continuously crossed with MY11-1 in an isolation field. Two primary translocation lines were selected from the progeny population. In southwestern China, MY11-1 is highly susceptible to stripe rust and powdery mildew. The T1RS.1BL translocation cultivar Chuan-nong10 (CN10), which inherited its 1RS chromosome from Petkus rye, was used as the control.

### Identification of Chromosomes

Chromosome construction of RT828-10 and RT828-11 were identified by multi-color fluorescence *in situ* hybridization (MC-FISH). Three probes, genomic DNA of Weining rye, pAs1, and pSc119.2 were used in the first MC-FISH experiment. The clone 6c6 is a wheat-specific centromeric sequence, and the clone pMD-CEN-3 is a rye-specific centromeric sequence. These 2 probes were used in the second MC-FISH experiment to identify the centromere structure. Meanwhile, sequence CCCTAAACCCTAAACCCTAAACCCTAAA was used as a probe to identify telomeres. The labeling processes of probes and *in situ* hybridization were conducted according to Fu et al. (2013) and Tang et al. (2014a,b). Images were captured using an epifluorescence microscope (model BX51, Olympus, Center Valley, PA, USA) equipped with a cooled charge-coupled device camera, and operated with the software program HCIMAGE Live (version 2.0.1.5, Hamamatsu Corp., Sewickely, PA, USA).

### Molecular Analysis

Total genomic DNA of 2 lines were isolated from young leaves by the surfactant cetyltrimethylammonium bromide. The seeds of RT828-10 and RT828-11 used in this experiment were collected from three different generations. O11B3 (5′-ggtaccaacaacaacaaccc-3′) and O11B5 (5′-gttgctgctgaggttggttc-3′) were used to detect the Glu-B3 gene on 1BS (Van Campenhout et al., 1995). ω-sec-P1 (5′-accttcctcatctttgtcct-3′) and ω-sec-P2 (5′-ccgatgcctataccactact-3′) were used to detect the Sec-1 gene on 1RS (Chai et al., 2006). The primer Gil-B1 (5′-gcagacctgtgtcattggtc-3′, 5′-gatatagtggcagcaggatacg-3′) was also used to detect the 1BS chromosome arms (Zhang et al., 2003). Furthermore, PrCEN-2 (5′-aatgatcttccacgacgacg-3′, 5′-cctcgttgggaaatggtgca-3′) was designed according to nucleotides 1140–2090 of the pAWRC.1 sequence (GenBank accession No. AF245032), which was used to amplify a rye-specific band and to analyze the structure of the centromere of the T1RS.1BL chromosomes (Li et al., 2016b).

PCR was carried out in a Bio-Rad iCycler thermal cycler (Bio-Rad Laboratories, Inc., Hercules, CA, USA). DNA was amplified with a 0.5 U Taq DNA polymerase enzyme, 1X buffer, 1.5 mM MgCl_2_, 200 μM dNTPs, 10 μmol primer, and 50 ng DNA in a total volume of 25 μL. After initial denaturation for 4 min at 94°C, each cycle included 60 s of denaturation at 94°C, 60 s of annealing at 60°C, and 2 min of extension at 72°C. A final extension for 10 min at 72°C followed the 30 cycles. PCR reactions were stored at 4°C until their resolution by electrophoresis on 1% agarose gels stained with ethidium bromide.

### Electrophoretic Detection of ω-Secalin Proteins

Detection of ω-secalin proteins by acid polyacrylamide gel electrophoresis (A-PAGE) was conducted as described by Li et al. (2016a) with minor modifications. The seeds were pulverized, and then the gliadin was extracted by 25% (v/v) ethylene chlorohydrin with 0.05% methyl green for 12 h at room temperature. The suspension was then centrifuged at 10,000 g for 10 min in a microfuge. Approximately 50 μL of the supernatant was collected and used for electrophoresis. Gliadin samples were loaded onto 2 mm thick 10% acrylamide gel, and were buffered with 0.5% (w/v) *N’N*-methylenebisacrylamide at pH 3.1. The gliadin was fractionated at 500 V for approximately 180 min. Then, gels were stained in 10% trichloroacetic acid (TCA) with 0.04% Coomassie Brilliant Blue G-250 and destained in 12% TCA.

### Resistance Analysis

RT828-10 and RT828-11 were examined for resistance to stripe rust and powdery mildew in the greenhouse and the field (Wan et al., 2004; Bariana and McIntosh, 1993). The *Pst* pathotypes CYR29 (virulent to *Yr1, 2, 3, 8, 9, 19, 23*), CYR31 (virulent to *Yr1, 2, 3, 6, 7, 9, 27*), CYR32 (virulent to *Yr1, 2, 3, 4, 6, 9, 27*), and CYR33 (virulent to *Yr1, 2, 3, 4, 6, 9, 15, 27*) were considered of high frequency and toxicity in southwestern China. Three other *Pst* pathotypes, SY5, SY6, and HY8, have been virulent to many released wheat cultivars in China in the past 2 decades, and are also virulent to *Yr9*. G22-9 is a new *Pst* pathogen and is prevalent in southwestern China, but in this experiment, all materials were resistant to it.

These 8 *Pst* pathotypes were used to inoculate the wheat plants. The *Pst* pathotypes were provided by the Plant Protection Institute, Gansu Academy of Agricultural Sciences, China. The *Bgt* isolate No. 9 was originally a single spore isolate culture collected in the field at Ya’an City, Sichuan, which was virulent to MY11-1, CN10 (*Pm8*), and Amigo (*Pm17*), and was used to inoculate RT828-10 and RT828-11 (Ren et al., 2009). Also, all plant materials were grown in the field and inoculated by mixed pathotypes of *Pst* and *Bgt* to determine the resistance response. Infection types (IT) of stripe rust were scored based on the 0–9 scale, as described by Wan et al. (2004). IT 0-3 are considered resistant, IT 4-6 are intermediate, and IT 7-9 are susceptible (Wan et al., 2004). The IT of powdery mildew was based on a 0–4 scale (Xie et al., 2004). Plants with an IT score of 0-2 were considered resistant, while those with an IT score of 3-4 were considered susceptible, according to Bariana and McIntosh (1993).

### Field Experiments for Determining Yield Components

All plants were grown in Qionglai District at Chengdu Plains, China in 2013–2015 following standard cultivation practices. Entries were arranged in a randomized, complete block design, in 3 m long plots, each consisting of four rows spaced 25 cm apart, at a plant density of 160 seedlings/m^2^ with three replications. Plant height and length of spikes of 10 randomly chosen plants were determined immediately before harvest. Samples for yield components were collected from each plot. Then, 1 m lengths from the center rows were cut to determine the number of spikes per square meter, 1,000-kernel weight, spikelet number per spike, kernel number per spike, and kernel weight per spike. Then, plots were harvested for grain yield analysis. Grain was weighed to obtain yield estimates based on 12% moisture and grain yield (kg/ha), including the grain weight from the 1 m sample (Kim et al., 2004; Ren et al., 2012). Fungicide was applied to the seedlings and again at heading to control diseases and pests.

### Statistical Analysis

Analysis of variance was performed for data of each character. The respective error term for the F-test was estimated using Sigmaplot 2001 software (SPSS Inc., Chicago, IL, USA). Least significant differences (LSD) were calculated for mean comparisons.

## Results

### Identification of the Chromosome Construction of Translocation Lines

**FIGURE 1:**
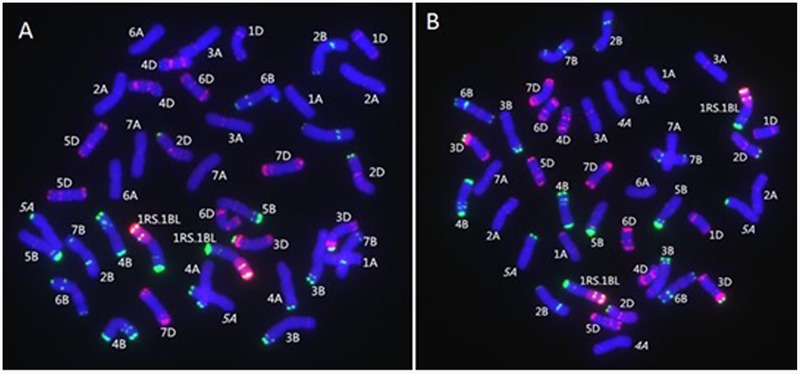
**MC-FISH of root tip chromosomes in wheat-rye translocation line RT828-10and RT828-11. (A)** RT828-10 contains a pair of T1RS.1BL translocation chromosomes. The signal patterns of pSc119.2 on the middle of 5A chromosome long arm were absence. **(B)** RT828-11 contains a pair of T1RS.1BL translocation chromosomes. The signal patterns of pSc119.2 on the middle of 5A chromosome long arm and the end of 4A chromosome long arm were absence. Rye genome DNA(red), pSc119.2-1(green), pAs1-1(red).

**FIGURE 2:**
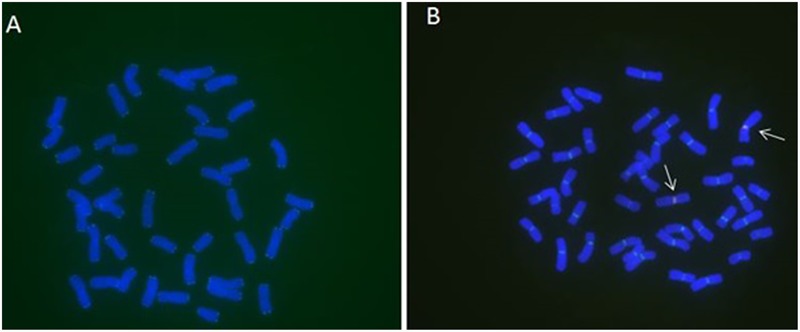
**MC-FISH of root tip chromosomes in wheat-rye translocation line RT828-10. (A)** The signal patterns of telomere of RT828-10. **(B)** The construction of centromere of RT828-10. The signal patterns of pMD-CEN-3 and 6c6 joint together in the centromere region. The arrows indicated the translocation chromosomes.pMD-CEN-3(red), 6c6(green).

**FIGURE 3:**
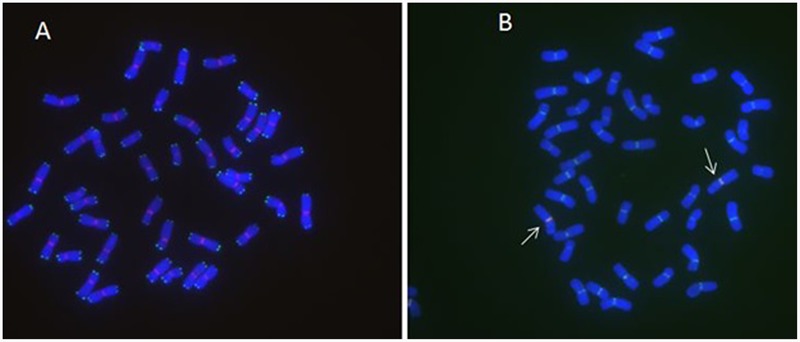
**MC-FISH of root tip chromosomes in wheat-rye translocation line RT828-11. (A)** The construction of telomere and centromere of RT828-11. 6c6(red), sequence CCCTAAACCCTAAACCCTAAACCCTAAA (green). **(B)** The construction of centromere of RT828-11. The signal patterns of pMD-CEN-3 and 6c6 joint together in the centromere region. The arrows indicated the translocation chromosomes.pMD-CEN-3(red), 6c6(green).

**FIGURE 4:**
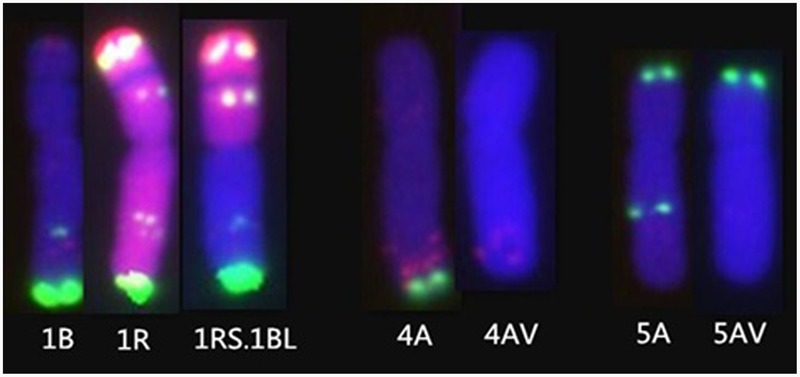
**The chromosome mutations of RT828-10 and RT828-11. (Left)** The FISH patterns of 1B chromosome of MY11, 1Rchromosome of Weining rye, and the T1RS.1BL translocation chromosome derived from MY11 X Weining. **(Middle)** 4A chromosome of MY11 and 4A chromosome of RT828-11. The signal patterns of pSc119.2 of the 4A chromosome long arm of RT828-11 are absence. **(Right)** 5A chromosome of MY11 and 5A chromosome of RT828-10 and RT828-11. The signal patterns of pSc119.2 of the 5A chromosome long arm are absence.

MC-FISH, PCR, and A-PAGE were used to identify the chromosome construction of lines RT828-10 and RT828-11. The results of MC-FISH (**Figures 1–3**) show that RT828-10 (2*n* = 42) and RT828-11 (2*n* = 42) contain an intact pair of wheat-rye T1RS.1BL translocation chromosomes. Moreover, compared with their parent MY11-1, the signal patterns of pSc119.2 of 5AL were absent in both RT828-10 and RT828-11, and the signal patterns of pSc119.2 of the 4AL were absent in RT828-11 (**Figures 1, 4**). Because the seeds used in the present study were collected from three different generations, the results of the MC-FISH indicated that RT828-10 and RT828-11 were cytogenetically stable, containing a pair of T1RS.1BL translocation chromosomes with several hereditable mutations on wheat chromosomes.

**FIGURE 5:**
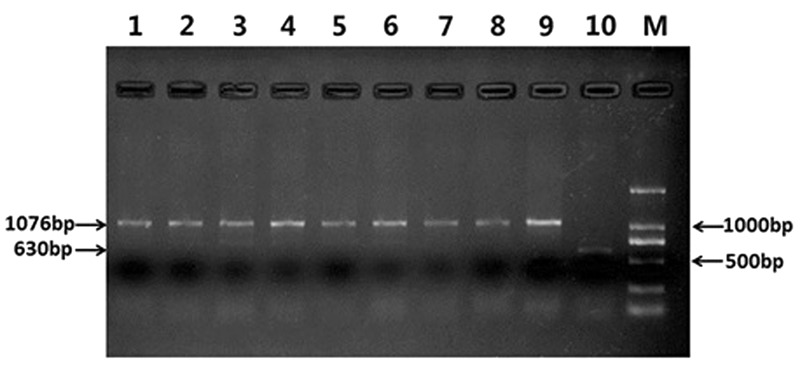
**Results of multiple-PCR by four specific primers: O11B3 and O11B5, ω-sec-P1 and ω-sec-P2.** Lane 1–4 = RT828-10 from different generations; Lane 5–8 = RT828-11 from different generations; lane 9 = CN10; lane 10 = MY11; lane M = marker.

Specific molecular markers were also used for identify the construction of RT828-10 and RT828-11. Primer pairs O11B3 and O11B5 could amplify a specific 630 bp fragment band from the wheat 1BS chromosome arm. On the other hand, primer pairs ω-sec-P1 and ω-sec-P2 could amplify a specific 1,076 bp fragment band from the rye 1RS chromosome arm. These 2 primers were used together in 1 PCR reaction. Common wheat could amplify only a 630 bp band, T1RS.1BL translocation lines could amplify only a 1,076 bp band, and heterozygous could amplify both bands. The PCR results indicated that only wheat parent MY11-1 could amplify a 630 bp band. All RT828-10 and RT828-11 lines collected from different generations could amplify a 1,076 bp band, but no 630 bp band was amplified (**Figure 5**).

**FIGURE 6:**
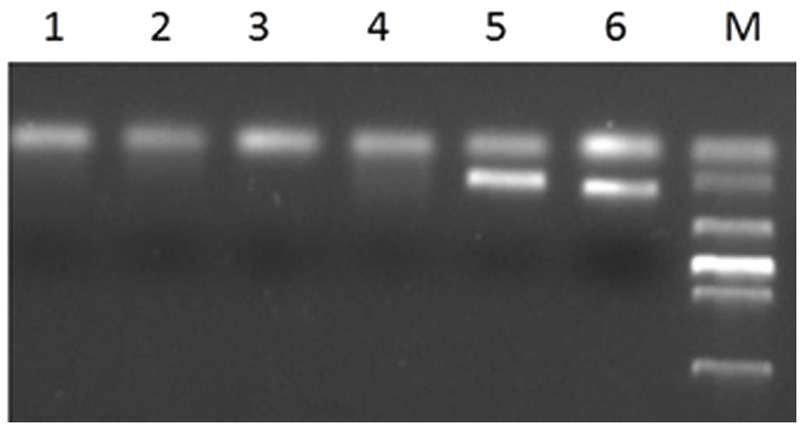
**PCR results of primer Gli-B1.** Lane 1–2 = RT828-10 from different generations; lane 3–4 = RT828-11 from different generations; lane 5 = Mianyang11; lane 6 = Chinese Spring; lane M = marker.

The primer Gli-B1 could also amplify a specific fragment band of wheat, about 220 bp. The PCR results showed that all translocation plants could not amplify the band with the expect size except the wheat parent MY11-1 and Chinese spring (**Figure 6**). It was indicated that the 1BS of lines RT828-10 and RT828-11 were absent.

**FIGURE 7:**
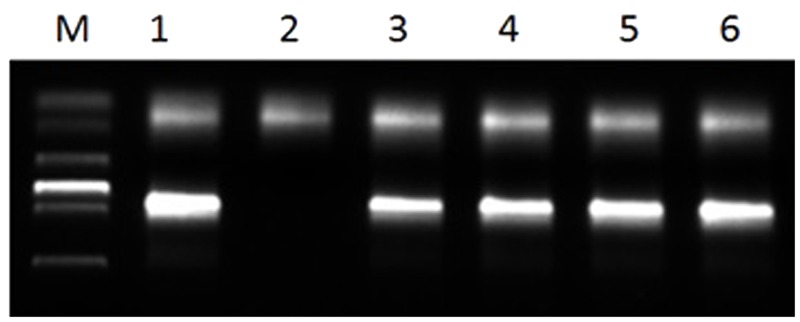
**PCR results of primer PrCEN-2.** Lane 1 = Weining Rye; lane 2 = MY11-1; lane 3–4 = RT828-10 from different generations; lane 5–6 = RT828-11 from different generations; lane M = marker.

The primer PrCEN-2 could amplify a specific fragment band about 1,000 bp of the rye centromere repetitive sequence, and was used to determine the breakpoint and the fusion point of the chromosome of the translocation lines. The PCR results showed that all translocation plants amplified a band with the expect size (**Figure 7**). It was also indicated that the translocation line RT828-10 and RT828-11 contained the full 1RS arm of rye.

**FIGURE 8:**
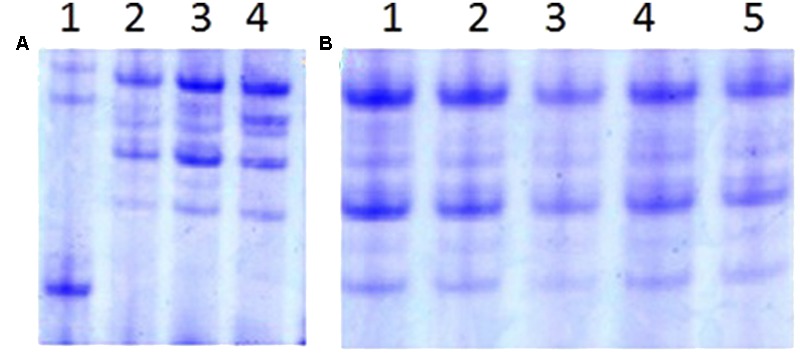
**A-PAGE separations of ω-secalins and gliadins from RT828-10 and RT828-11. (A)** Lane 1 = wheat parent MY11-1; lane 2–4 = RT828-10 from different generations; **(B)** Lane 1–5 = RT828-11 from different generations

The w-secalin, which was encoded by the Sec-1 locus in the 1RS chromosome arm, could be used as a biochemistry marker to identify the 1RS chromosome arm. The specific bands of w-secalin were investigated by A-PAGE. All RT828-10 and RT828-11 samples exhibited normal expression for the genes at the Sec-1 locus (**Figure 8**). It was also observed that RT828-10 and RT828-11 contained the 1RS chromosome arms.

### Analysis for Resistance to Stripe Rust and Powdery Mildew

Wheat parent MY11-1 was highly susceptible to 6 *Pst* pathotypes excluding HY8 and G22-9, while Weining rye was highly resistant to all *Pst* pathotypes (**Table 1**). Wheat cultivar CN10, whose 1RS chromosome arms came from the Russian wheat cultivar ‘Aurora’ (*Yr9*), were also highly susceptible to 5 *Pst* pathotypes except SY5, SY6, and G22-9 (**Table 1**). T1RS.1BL translocation line RT828-10 and RT828-11 showed high resistance to all *Pst* pathotypes (**Table 1**). On the other hand, MY11-1 and CN10 were highly susceptible to the *Bgt* isolate No. 9, but RT828-10 and RT828-11 showed high resistance to the isolate (**Table 1**). Moreover, RT828-10 and RT828-11 also showed high resistance to stripe rust and powdery mildew in the field (**Table 1**).

**Table 1:** Analysis of resistance to stripe rust in translocation lines and theirs’ wheat parent when inoculated with epidemic pathotypes and isolates of *Puccinia striiformis* f. sp. *Tritici* and *Blumeria graminis* f. sp. *tritici.*

	Pstpathotypes and isolates of PST									Isolates of Bgt	
Translocation lines, wheat parent and control	CYR29	CYR31	CYR32	CYR33	SY5	SY6	HY8	G22-9	In field	Isolate No.9	In field
RT828-10	0	0	0	0	0	0	0	0	0	0	0
RT828-11	0	0	0	0	0	0	0	0	0	0	0
MY11-1(wheat parent)	8	8	8	8	8	6	0	0	7	4	4
Chuan-Nong10(CK)	6	6	8	8	0	0	6	0	6	3	4
Weining rye	0	0	0	0	0	0	0	0	0	0	0

*Sripe rust severities are recorded on an infection type scale of 0–9 as described by Wan et al. (2004). where, 0 = immune, no visible symptoms; 1–3 = resistant, increasing from (i) flecks, no necrosis to (ii) necrosis, to (iii) chlorotic areas with slight sporulation; 4–6 = intermediate, chlorotic areas decreasing in amount while mycelium and conidial production increases from slight to moderate; 7–9 = susceptible, increasing amount, size, and density of mycelium and conidia to a fully compatible reaction. Powdery mildew severities are recorded on an infection type scale of 0–4 as described by Bariana and McIntosh (1993) and Xie et al. (2004), where, 0–2 = resistant, 0 = no visible symptoms and signs, 0 = necrotic flecks without sporulation, 1 = small uredia with necrosis, 2 = small to medium sized uredia with necrotic spots; 3–4 were considered susceptible: 3 = medium sized uredia with or without chlorosis, 4 = large uredia without chlorosis.*

### Effect of Chromosome Translocation on Agronomic Traits of Wheat

Translocation lines RT828-10 and RT828-11 were derived from the same monosomic addition plant (2n = 43 = 21″W + 1R), which originated from wheat MY11-1 and Weining rye. Compared with MY11-1, both translocation lines showed good agronomic traits. Specifically, in RT828-11, a significant increase (*P* < 0.05) was observed for plant height, spikelet number per spike, kernel number per spike, kernel weight per spike, 1,000-kernel weight, number of spikes per square meter, and grain yield (**Table 2**). Compared with MY11-1, RT828-10 showed significantly lower kernel weight per spike and 1,000-kernel weight. However, the grain yield of RT828-10 was still higher than that of MY11-1 because of the significantly higher number of spikes per square meter. The variant translocation line RT828-11, which was lack of the pSc119.2 signal patterns of 4AL and 5AL (**Figure 4**), showed significantly better agronomic traits than another translocation line, RT828-10, which lacks the pSc119.2 signal of 5AL (**Figure 4**). The plant height, spikelet number per spike, kernel number per spike, kernel weight per spike, 1,000-kernel weight, number of spikes per square meter and grain yield of RT828-11 were significantly higher than that of RT828-10 (**Table 2**). These results indicated that there are positive effects of the variant chromosome on agronomic traits.

**Table 2:** Comparisons of agronomic traits among RT828-10, RT828-11 and theirs’ wheat parent.

Lsines	PH (cm)	SLN (per spike)	KN (per spike)	KW (per spike)	TKW (g)	NS (m^-2^)	GY (kg/ha)	LS (cm)
RT828-10	87.3a	20.6a	47.8a	1.83a	38.3a	356.2a	6530.3a	8.9a
RT828-11	111.4b	23.1b	54.1b	2.58c	47.7c	332.3b	8578.5b	9.4ab
Mianyang 11(wheat)	85.8a	20.8a	47.9a	2.16b	45.0b	300.8c	6484.8a	10.1b

*PH = plant height, SLN = spikelet number per spike, KN = kernel number per spike, KW = kernel weight per spike, TKW = 1,000-kernel weight, NS = number of spikes per square meter, GY = grain yield. LS = length of spike. Values with the same letter in the same column do not differ significantly at *P* < 0.05.*

## Discussion

### Development and Identification of Variant Wheat-rye Translocation Lines

T1RS.1BL translocation lines are not only important genetic resources for wheat breeding programs, but also materials for the study of genetics, physiology, and phytopathology of polyploidy plants (Eckardt, 2010; Wicker et al., 2003; De Storme and Mason, 2014). In the present study, 2 new primary T1RS.1BL translocation lines were developed from the progeny of the cross of rye variety Weining and wheat cultivar MY11-1. Multiple methods were used to screen the homozygote T1RS.1BL translocation lines from the enormous amount of progeny of the cross. A-PAGE was able to identify the w-secalin encoded by the Sec-1 locus of 1RS chromosome arm, and w-secalin was considered as a biochemistry marker of the 1RS chromosome arm. Thus, A-PAGE could be used as a tool to screen the presence of 1RS chromosome arms in the progeny of wheat–rye crossings with enormous amounts of seeds. Although A-PAGE is efficient, it could not identify the existence form of the 1RS chromosome arms in the wheat genetic background. Moreover, several new T1RS.1BL translocation lines were expression deletion of gene Sec-1, and A-PAGE could not screen these particular T1RS.1BL translocation lines (Li et al., 2016a). C-banding and genomic *in situ* hybridization (GISH) have also been used to identify rye chromosomes in wheat genetic backgrounds. GISH showed that there were several rye chromatins in the wheat genome, but could not identify which rye chromosomes were introduced into the wheat genome. C-banding could identify most of the chromosomes of rye and wheat, but it was too difficult to conduct C-banding and obtain high resolution pictures. In this study, MC-FISH was used to confirm the construction of wheat lines RT828-10 and RT828-11.

The probe pAs1 can identify the D-genome chromosomes and was able to show different signal patterns on 1A, 2A, 3A, 4A, 6A, 7A, 1B, 2B, 3B, 6B, and 7B chromosomes of wheat (Schneider et al., 2003; Tang et al., 2014a,b). The probe pSc119.2 could identify the B-genome and rye chromosomes, and also showed different signal patterns on the 2A, 4A, 5A, 2D, 3D, and 4D chromosomes of wheat (Schneider et al., 2003; Contento et al., 2005; Tang et al., 2014a,b). Multiple probes were used in MC-FISH. Furthermore, the mixed probes of pAs1, pSc119.2, and rye genomic DNA could directly distinguish different wheat and rye chromosomes in 1 cell (Tang et al., 2014a,b; Ren et al., 2016). Compared with FISH or C-banding, MC-FISH was much more efficient and precise. Our MC-FISH results indicated that both lines were T1RS.1BL translocation lines. It also showed that both lines lacked the signal patterns of pSc119.2 of 5AL, and that RT828-11 simultaneously lacked the signal patterns of pSc119.2 of 4AL. These chromosome mutants could be induced during the progress of chromosome translocation. The occurrence of genes or DNA fragment mutants induced with the chromosomal variation, including translocation, substitution, addition, and polyploidization, can be considered accompanying mutations (Li et al., 2016a), and the accompanying mutations of the evolutionarily significant translocations are remarkable resources for plant improvement.

Moreover, molecular markers were used to identify translocation chromosomes. Several molecular markers were developed, and were specific for an individual locus or genes of wheat or rye, such as O11B3/B5 and ω-sec-P1/P2 (Van Campenhout et al., 1995; Chai et al., 2006). It was efficient to use these markers to detect rye chromatin in the wheat genome. In the present study, our results indicated that RT828-10 and RT828-11 are stable new primary T1RS.1BL translocation lines.

Tang et al. (2009) indicated that allopolyploidization could induce immediate microsatellite evolution, and Li et al. (2016a) reported that the *Sec-1* gene, which is located on 1RS, was mutant after chromosome translocation. Additionally, Tang et al. (2014b) reported that the signal patterns of pSc119.2 of 5AL were mutant after polyploidization. It was suggested that the mutant of chromosome structure could be induced by polyploidization and translocation. In the present study, compared with the wheat parent MY11-1, the signal patterns of pSc119.2 of 5AL were mutant in both translocation lines, and signal patterns of 4AL were also mutant in RT828-11 (**Figures 1, 4**).

### Resistance to Stripe Rust and Powdery Mildew

Hundreds of wheat cultivars have been released from the globally used T1RS.1BL translocation line Aurora, which originated from Petkus rye (Rabinovich, 1998). Therefore, the 1RS chromosome arms lacked genetic diversity (Schlegel and Korzun, 1997; Ren et al., 2009, 2012). Since the 1990s, due to the prevalence of virulent pathotypes, virulence to *Yr9* and *Pm8* have not provided protection to the respective pathogens anymore (Ren et al., 2009). Several scientists tried to transfer new sources of 1RS chromosomes of rye into common wheat. These new T1RS.1BL translocation lines showed diversity-resistant patterns to rust stripe and powdery mildew. However, few new translocation lines showed both high resistance to these two diseases and could be used in the wheat breeding program (Ko et al., 2002; Yang et al., 2014; Li et al., 2016b; Qi et al., 2016).

Rye is a cross-pollinated plant with high genetic diversity in the population of a variety. It was suggested that the abundance of variations could be consist in rye varieties (Ren et al., 2011). In the present study, 2 new stable primary T1RS.1BL translocation lines, RT828-10 and RT828-11, derived from Chinese rye variety Weining, showed high resistance to 8 *Pst* pathotypes that are prevalent in China. RT828-10 and RT828-11 also showed high resistance to *Bgt* isolate, which is virulent to *Pm8* and *Pm17*. Furthermore, RT828-10 and RT828-11 exhibited resistance to the prevalent pathotypes to these two diseases more than the T1RS.1BL translocation cultivar CN10, which harbors genes *Yr9* and *Pm8*. Since the wheat parent MY11-1 was highly susceptible to stripe rust and powdery mildew, it is obvious that the resistant genes in RT828-10 and RT828-11 must be located on the 1RS chromosome arms, which were derived from Weining rye. The new resistance genes provided better protection to diseases than *Yr9, Pm8*, and *Pm17.*

### The Effect of Agronomic Traits of Variation Translocation Lines

Both translocation lines showed good agronomic traits, especially RT828-11, which showed a significant increase over its wheat parent MY11-1. Although RT828-10 and RT828-11 were T1RS.1BL translocation lines with variation, they had a normal phenotype in the field, similar to their wheat parent and other T1RS.1BL translocation lines also derived from MY11-1. The genetic effects of chromosome variation could be detected by comparing these 2 lines with wheat parent MY11-1. The 1RS.1BL translocations were considered favorable for agronomic performance (Villareal et al., 1995; Ren et al., 2012); however, the effects of the 1RS.1BL translocation were not consistent for grain yield. Several studies reported no significant effect on grain yield in 1RS.1BL translocation lines (McKendry et al., 1996; Singh et al., 1998), but in another study, the 1RS source was suggested to be responsible for yield enhancement (Kim et al., 2004) because significant interactions between wheat and rye sources existed for grain yield (Jung and Lelley, 1985; Ren et al., 2012). The 1RS.1BL translocations increased grain yield only in specific combinations of 1RS and 1BL. In the present study, we found that only RT828-11 remarkably increased the agronomic performance. Introduction of the 1RS arm significantly improved several agronomic traits in RT828-11, such as PH, SLN, KN, KW, TKW, NS, and GY (**Table 2**).

## Author Contributions

TR and ZL designed this work. TR, ZT, SF did the MC-FISH. TR, ZL did the molecular analyze and A-PAGE. TR, ZR did the resistance analyze. TR, ZR, BY, FT did the field work. TR and ZL wrote the manuscript.

## Conflict of Interest Statement

The authors declare that the research was conducted in the absence of any commercial or financial relationships that could be construed as a potential conflict of interest. The reviewer AB and handling Editor declared their shared affiliation, and the handling Editor states that the process nevertheless met the standards of a fair and objective review.
